# A pilot study on a potential relationship between leg bone length and sprint performance in sprinters; are there any event-related differences in 100-m and 400-m sprints?

**DOI:** 10.1186/s13104-020-05140-z

**Published:** 2020-06-22

**Authors:** Daichi Tomita, Tadashi Suga, Masafumi Terada, Takahiro Tanaka, Yuto Miyake, Hiromasa Ueno, Mitsuo Otsuka, Akinori Nagano, Tadao Isaka

**Affiliations:** 1 Faculty of Sport and Health Science, Ritsumeikan University, 1-1-1 Nojihigashi, Kusatsu, Shiga 525-8577 Japan; 2 Graduate School of Health and Sport Science, Nippon Sport Science University, Tokyo, Japan; 3 Research Fellow of Japan Society for the Promotion of Science, Tokyo, Japan

**Keywords:** Femoral length, Tibial length, Step frequency, Step length, Magnetic resonance imaging

## Abstract

**Objective:**

This study examined the relationship between leg bone length and sprint performance in sprinters. The leg bone lengths in 28 100-m specialized sprinters and 28 400-m specialized sprinters were measured using magnetic resonance imaging. The lengths of the upper and lower leg bones were assessed by calculating the lengths of the femur and tibia, respectively. To minimize differences in body size among participants, both bone lengths were normalized to body height. The ratio of the tibial length to femoral length was calculated to evaluate the interaction between the lengths of the upper and lower leg bones. International Amateur Athletic Federation (IAAF) scores, based on the personal best times of the sprinters in each group were used as parameters for sprint performance.

**Results:**

There were no significant correlations between absolute and relative lengths of the femur and tibia and IAAF scores in both 100-m and 400-m sprinters. By contrast, the ratio of the tibial length to femoral length correlated significantly with IAAF score in 400-m sprinters (*r* = 0.445, *P* = 0.018), but not 100-m sprinters. These findings suggest that the leg bone lengths may play an important role in achieving superior long sprint performance in 400-m specialized sprinters.

## Introduction

Some morphological factors are associated with superior sprint performance in sprinters [1–7]. Sprint velocity is expressed as the product of step length and frequency [8], suggesting that morphological factors regulating the two sprint variables may play important roles in achieving superior sprint performance in sprinters. The leg length is a major morphological factor regulating step length [9, 10]; thus, longer leg may result in better sprint performance due to an increased step length during sprinting [11]. We and others previously found a positive relationship between leg length and running performance in endurance runners [12–14]. However, the effect of leg length on sprint performance in sprinters is still poorly understood.

Previous studies reported that step frequency may be a more important kinematic factor for superior sprint performance than step length [15–18]. Morin et al. [15] demonstrated a positive correlation between step frequency and sprint velocity during 100-m sprinting; however, no such correlation was obtained with step length. Hobara et al. [16] reported that step frequency correlated with vertical stiffness (i.e., the ratio of peak vertical force and vertical center of mass displacement), which is positively related to sprint velocity, during 400-m sprinting. However, no such correlation was also obtained with step length. Therefore, morphological factors regulating step frequency may be more important in achieving superior sprint performance than that regulating step length.

Physiological factors, including anaerobic capacity and fatigue resistance, are known to play important roles in achieving superior long sprint performance during 400-m sprinting [19, 20]. Utilizing these physiological capacities during a 400-m sprint can be mitigated by sprinting economically. The leg is swung forward with the knee bent during the swing phase while sprinting. Based on this leg behavior, an increased ratio of the lower leg length relative to the upper leg length (i.e., thigh length) may reduce the leg’s moment of inertia and positive work done by the hip flexors during the swing phase, potentially due to decreased leg mass, since the lower leg has less mass than that of the upper leg [21]. Therefore, this favorable morphology may be useful in enhancing step frequency and performing economical sprinting. Considering these findings, we hypothesized that a higher ratio of the lower leg length to upper leg length would be positively related to better sprint performance in sprinters, especially in 400-m specialized sprinters.

Generally, the leg length is measured with an anthropometrical technique, which is performed manually using a tape measure. Nevertheless, this measurement may have a technical limitation due to possible ambiguity in measuring the leg length. Compared to this general anthropometrical measurement, magnetic resonance imaging (MRI) is more appropriate for morphological measurements [1–7, 14, 22], including bone length measurements [4, 5, 14, 22]. To test our hypothesis with higher precision, we used MRI to examine the relationships between leg bone length variables and sprint performance in both 100-m and 400-m specialized sprinters.

## Main text

### Methods

#### Subjects

Fifty-six male sprinters participated in this study. Of those, 28 sprinters (age: 20.3 ± 1.6 years) specialized in the 100-m race and 28 sprinters (age: 20.1 ± 1.4 years) specialized in the 400-m race. Sprinters in each group were well-trained and competed regularly at their distances. Mean personal best time for the 100-m specialized sprinters was 11.05 ± 0.33 s. Mean personal best time for the 400-m specialized sprinters was 49.96 ± 1.63 s. Mean International Amateur Athletic Federation scores (an interindividual score for the comparison of competitive performances in different events) based on these personal best time were used as parameters for sprint performance in each group. Informed written consent was obtained from all participants. This study was approved by the Ethics Committee of Ritsumeikan University.

#### MRI measurements

**Fig. 1:**
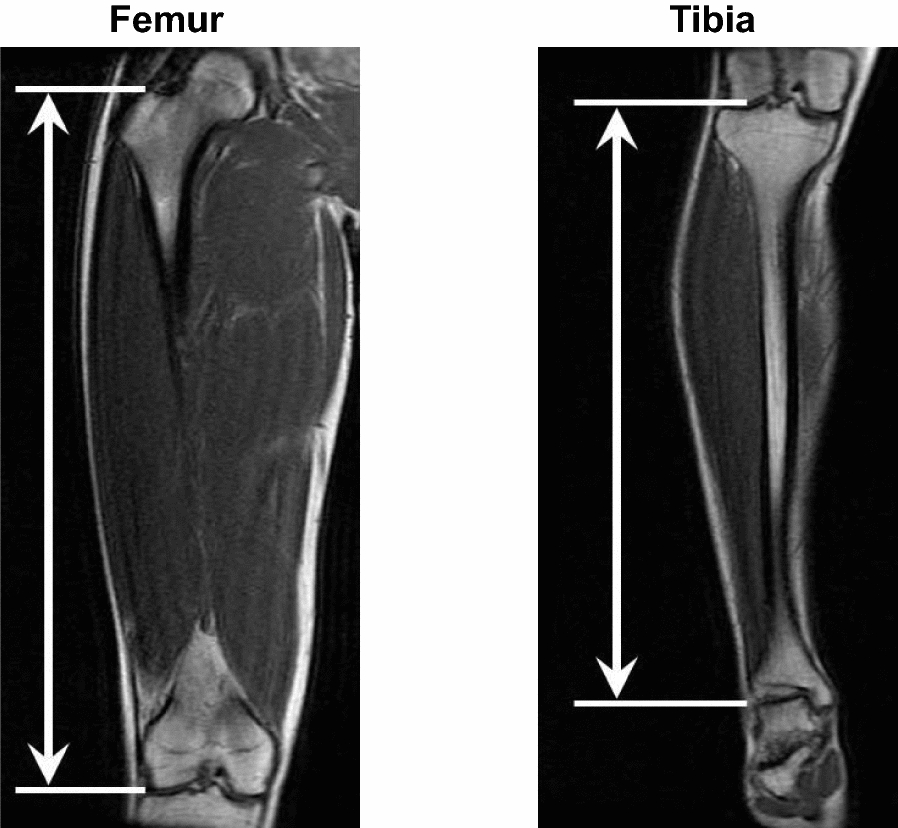
Representative magnetic resonance imaging scans used for measuring the lengths of the femur and tibia. The femoral length was measured as the distance between the tip of the greater trochanter and the distal end of the lateral condyle of the femur. The tibial length was measured as the distance between the proximal end of the lateral condyle and the distal inferior surface of the tibia

Representative images for calculating the leg bone lengths on magnetic resonance imaging (MRI) are shown in Fig. 1. The MRI measurement has been previously described [14]. The analyses for measuring the lengths of the leg bones were conducted using image analysis software (OsiriX Version 5.6; OsiriX Foundation, Geneva, Switzerland). The lengths of the femur and tibia were calculated from coronal images for the leg, respectively. The femoral length was calculated as the distance between the tip of the greater trochanter and the distal end of the lateral condyle of the femur. The tibial length was calculated as the distance between the proximal end of the lateral condyle and the distal inferior surface of the tibia. The total length of the femur and tibia was calculated to assess the overall leg bone length. To minimize differences in body size among participants, these both lengths were normalized to body height. The ratio of the tibial length to femoral length was calculated to evaluate interaction between the lengths of the upper and lower leg bones. The reproducibility of the leg bone length has been noted in our previous study [14].

#### Statistical analysis

The data are presented as the mean ± SD. Comparisons of groups were performed using an unpaired *t*-test. Relationship between variables was evaluated using a Pearson’s product moment correlation. Statistical significance was defined at *P* < 0.05. All statistical analyses were conducted using IBM SPSS software (version 19.0; International Business Machines Corp, NY, USA).

### Results

IAAF scores did not differ significantly between 100-m specialized sprinters and 400-m specialized sprinters (873 ± 97 and 863 ± 97).

Physical characteristics and leg bone length variables of the 100-m and 400-m sprinters are summarized in Table 1. Physical characteristics (i.e., body height, body weight, and body mass index) did not differ between 100-m and 400-m sprinters. Additionally, all leg bone length variables did not differ between the two groups.

**Table 1:** Physical characteristics and leg bone length variables in 100-m and 400-m specialized sprinters

	100-m sprinters	400-m sprinters	*F*	*P*	Cohen’s *d*
Body height, cm	173.2 ± 4.0	172.4 ± 4.1	0.088	0.437	0.198
Body weight, kg	64.6 ± 5.2	62.8 ± 4.1	1.462	0.173	0.384
Body mass index, kg/m^2^	21.5 ± 1.3	21.1 ± 0.9	3.925	0.232	0.358
Leg bone length					
Femur, mm	432.0 ± 15.6	430.8 ±v12.2	1.588	0.737	0.086
Tibia, mm	359.7 ± 14.7	357.9 ± 13.0	1.678	0.620	0.130
Relative leg bone length					
Femur, % of body height	24.9 ± 0.6	25.0 ± 0.4	3.868	0.696	0.196
Tibia, % of body height	20.8 ± 0.6	20.8 ± 0.6	0.210	0.984	0.000
Total leg bone length					
Femur + Tibia, mm	791.7 ± 29.5	788.6 ± 23.2	2.311	0.663	0.117
Relative total leg bone length					
Femur + Tibia, % of body height	45.7 ± 1.1	45.7 ± 0.8	3.466	0.850	0.000
Ratio between leg bones					
Tibia/Femur, mm/mm	0.83 ± 0.02	0.83 ± 0.02	3.626	0.670	0.000

Values are presented as Mean ± SD

Coefficient correlations between leg bone length variables and IAAF score of the 100-m and 400-m sprinters are summarized in Table 2. Absolute and relative lengths of the femur and tibia did not correlate with IAAF scores in both 100-m and 400-m sprinters. Absolute and relative total lengths of the femur and tibia also did not correlate with IAAF Score in the two groups. By contrast, the ratio of the tibia length to femur length correlated with IAAF score in 400-m sprinters, but not in 100-m sprinters.

**Table 2:** Correlation coefficients between leg bone length variables and sprint performance (International Amateur Athletic Federation score) in 100-m and 400-m specialized sprinters

	100-m sprinters		400-m sprinters	
	*R* [lower limit, upper limit]	*P* value	*R* [lower limit, upper limit]	*P* value
Leg bone length				
Femur	− 0.166 [− 0.508, 0.221]	0.399	0.007 [− 0.367, 0.379]	0.970
Tibia	− 0.173 [− 0.513, 0.214]	0.380	0.336 [− 0.042, 0.630]	0.081
Relative leg bone length				
Femur	0.004 [− 0.370, 0.377]	0.983	− 0.273 [− 0.586, 0.111]	0.160
Tibia	− 0.036 [− 0.404, 0.342]	0.857	0.257 [− 0.128, 0.575]	0.187
Total bone length				
Femur + Tibia	− 0.174 [− 0.514, 0.213]	0.376	0.191 [− 0.196, 0.527]	0.330
Relative total leg bone length				
Femur + Tibia	− 0.016 [− 0.387, 0.359]	0.935	0.040 [− 0.338, 0.407]	0.841
Ratio between leg bone lengths				
Tibia/Femur	− 0.017 [− 0.388, 0.358]	0.932	*0.445 [0.086, 0.702]*	*0.018*

International Amateur Athletic Federation scores, based on the personal best times of each event for 100-m and 400-m specialized sprinters were used as parameters for sprint performance. Italic values indicate a significant correlation (*P* < 0.05) between leg bone length variable and sprint performance in 400-m specialized sprinters

### Discussion

The primary finding of this study was that a higher ratio of the tibia length to femoral length correlated with better IAAF score in 400-m sprinters, but not in 100-m sprinters. Maintaining a consistent step frequency during 400-m sprinting is required for achieving superior long sprint performance [16, 23]. Morphological factors may contribute to the maintenance of step frequency, potentially by sustaining economical sprinting [5, 6]. The ratio between the leg bone lengths may be useful in reducing the leg’s moment of inertia and the positive work of the hip flexors during the swing phase while sprinting. Thus, this favorable morphology may help achieve superior long sprint performance, potentially by maintaining step frequency and allowing for economical sprinting in 400-m specialized sprinters.

This study showed no correlation between the ratio of the tibia length to femur length and IAAF score in 100-m sprinters. In general, superior 100-m sprint performance may not require economical movement, because sprint velocity during 100-m sprinting does not decrease significantly compared to that during 400-m sprinting [11, 24]. Furthermore, superior 100-m sprint performance is related to greater ground reaction force during 100-m sprinting [15, 40]. An increase in the ground reaction force during 100-m sprinting may be associated with greater sizes of some leg muscles because of the positive relationships between these muscle sizes and 100-m sprint performance [1, 3, 7]. In particular, previous studies determined that greater thigh muscles, including the quadriceps femoris and hamstring, correlated with better 100-m sprint performance in sprinters [1, 3, 7]. When having a higher ratio of the tibial length to femoral length in 100-m sprinters, this morphology may be modeling smaller thigh muscles due to a necessary shortening of the thigh length. Therefore, the ratio between the leg bone lengths may not relate to 100-m sprint performance in 100-m specialized sprinters.

We previously reported using MRI that relative lengths of the leg bones (i.e., tibial length and total length of the femur and tibia) normalized to body height correlated with running performance in endurance runners [14]. By contrast, in the present study, absolute and relative individual and total lengths of the leg bones did not correlate with sprint performance in either 100-m or 400-m sprinters. Although the longer leg is associated with an increase in step length during sprinting [9, 10], step length may be a less important kinematic factor for superior sprint performance than step frequency during both 100-m and 400-m sprinting [15–17]; specifically, an increase in step length is not required for achieving superior sprint performance. Only one study by Morin et al. [15] reported that relative length of the leg (i.e., the distance from the great trochanter to the ground) normalized to body height did not correlate with 100-m sprint velocity. Therefore, the present findings corroborate with this result by showing an absence of a relationship between the leg length and sprint performance in 100-m specialized sprinters. Furthermore, the present study is the first to determine that longer leg may not be required for achieving superior long sprint performance in 400-m specialized sprinters.

This study showed that all bone length variables did not differ between 100-m and 400-m sprinters; thus, characteristics of the leg bone length are similar between the two groups. In additional analyses of the present study, absolute total and individual lengths of the femur and tibia in sprinters (a combined group of 100-m and 400-m sprinters) were higher than those in 5000-m endurance runners (e.g., 431.4 ± 13.9 vs. 420.8 ± 20.2 mm for the femoral length and 358.8 ± 13.8 vs. 351.3 ± 18.2 mm for the tibial length; *P* < 0.05 for both) observed in our previous study [14], which may be due to greater body height for sprinters than that for endurance runners (172.8 ± 4.0 vs. 169.6 ± 5.6 cm, *P* = 0.01). By contrast, relative individual and total lengths of the femur and tibia and a ratio of the tibial length to femoral length did not differ between sprinters and endurance runners (25.0 ± 0.4 vs. 24.8 ± 0.7% of both height for the relative femoral length, 20.8 ± 0.6 vs. 20.7 ± 0.6% of body height for the relative tibial length, 0.83 ± 0.02 vs. 0.84 ± 0.02; *P* > 0.05 for all). Similar results were also observed for the leg bone length variables between 100-m and 400-m sprinters and endurance runners (data not shown). Therefore, characteristics of the leg bone lengths relative to body size and the ratio between the leg bone lengths may be similar among athletes competing in distances from 100-m to 5000-m.

Our previous studies determined that longer forefoot bones and greater knee extensor moment arm correlated with better long sprint performance in 400-m sprinters [5, 6]. To the best of our knowledge, no other researchers have reported morphological factors for superior long sprint performance in 400-m sprinters. These favorable morphological factors of superior 400-m sprint performance obtained in our previous studies are also determinants for achieving superior 100-m sprint performance [2, 4]. By contrast, a higher ratio of the tibia length to femoral length may be a determinant only of 400-m sprinters. Therefore, the present study is the first to find a specific morphological factor contributing to superior long sprint performance in 400-m specialized sprinters. The information may be helpful to selecting sprint events and understanding individual features in sprinters, particularly in 400-m specialized sprinters.

## Limitations

We hypothesized that a higher ratio of the tibial length relative to femoral length may help achieve superior long sprint performance, potentially by maintaining step frequency and performing economical sprinting in 400-m specialized sprinters. However, we did not measure kinetic (e.g., ground reaction force) and kinematic (e.g., step frequency) data during 400-m sprinting. Further studies are needed to examine the relationships between the leg bone length variables and kinetic and kinematic data during 400-m sprinting.

## Data Availability

Data will be provided the corresponding author upon request.

## Abbreviations

IAAF: International Amateur Athletic Federation
MRI: Magnetic resonance imaging
